# Cl^−^ Doping Strategy to Boost the Lithium Storage Performance of Lithium Titanium Phosphate

**DOI:** 10.3389/fchem.2020.00349

**Published:** 2020-05-12

**Authors:** Hao Luo, Yijun Tang, Zeying Xiang, Pinghui Wu, Zhizhong Li

**Affiliations:** ^1^School of Science, Southwest University of Science and Technology, Mianyang, China; ^2^College of Science, Zhejiang University of Technology, Hangzhou, China; ^3^Research Center for Photonic Technology, Fujian Key Laboratory for Advanced Micro-Nano Photonics Technology and Devices & Key Laboratory of Information Functional Material for Fujian Higher Education, Quanzhou Normal University, Fujian, China; ^4^Basic Teaching Department, Neusoft Institute Guangdong, Foshan, China

**Keywords:** aqueous rechargeable lithium batteries, anode, anion doping, lithium titanium phosphate, electrochemical performance

## Abstract

Because of energy storage limitations and the high demand for energy, aqueous rechargeable lithium batteries (ARLBs) are receiving widespread attention due to their excellent performance and high safety. Lithium titanium phosphate (LiTi_2_(PO_4_)_3_) exhibits the potential to serve as anodes for ARLBs because it has a three-dimensional channel and a stable structure. We employed an anion (Cl^−^) doping strategy to boost the lithium storage performance of LiTi_2_(PO_4_)_3_. A series of LiTi_2_(PO_4_)_3_/C composites doped with Cl^−^ on ${\text{PO}}_{4}^{3-}$ were successfully synthesized with a sol-gel technique as anodes for ARLBs. The effects of chlorine doping with different content on the properties of LiTi_2_(PO_4_)_3−*x*_Cl_3*x*_/C (*x* = 0.05, 0.10, and 0.15) were investigated systematically. The doping of chlorine in appropriate amounts did not significantly impact the main structure and morphology of LiTi_2_(PO_4_)_3_/C. However, chlorine doping greatly increased the performance of LiTi_2_(PO_4_)_3_/C. LiTi_2_(PO_4_)_2.9_Cl_0.3_/C (LCl-10) showed the best electrochemical properties. It delivered a discharge capacity of 108.5 and 85.5 mAh g^−1^ at 0.5 and 15°C, respectively, with an increase of 13.2 and 43.3 mAh g^−1^ compared to blank LiTi_2_(PO_4_)_3_ (LCl). In addition, the discharge capacity of LCl-10 was maintained at 61.3% after 1,000 cycles at 5°C, implying an apparent improvement compared to LCl (35.3%). Our study showed that a chlorine-doped LiTi_2_(PO_4_)_3_/C composite is a potential anode for high-performance ARLBs.

## Introduction

With the advancement of modern society, environmental pollution, and energy shortages have become more serious (He et al., 2019; Jiang et al., 2019; Zhou et al., 2019; Huan et al., 2020; Li et al., 2020a,b). Electrical energy can be derived from various other forms of energy, however, as an effective means to solve these problems. The secondary battery system is a necessary device for energy conversion and storage (Wu et al., 2017; Fang et al., 2019; Yang et al., 2019; Lu et al., 2020). The needs of high-performance electronic devices and electric vehicles have motivated the rapid development of lithium-ion batteries (LIBs) in recent years. The demand for LIBs offering high energy density, low pollution, and long life has rapidly increased (Wang et al., 2012; Zhao et al., 2016; Hua et al., 2018; Zhang et al., 2020). Conventional LIBs typically use organic electrolytes, which have demonstrated poor rate performance and potential safety hazards. ARLBs can efficiently solve these problems because they are environmentally friendly, highly secure, and inexpensive (Zhu et al., 2016; Lakhnot et al., 2019). Indeed, many cathodes of ARLBs exhibit remarkable electrochemical performance, such as LiMn_2_O_4_ (Manjunatha et al., 2011), LiFePO_4_ (He et al., 2011), and LiCoO_2_ (Ruffo et al., 2011).

Compared to cathodes, the restricted performance of anodes cannot meet the need for high-performance from ARLBs. Excellent anode materials are a limiting factor for the development of ARLB. Currently, vanadium oxide [VO_2_ (Li et al., 1994)] and vanadate [LiV_3_O_8_ (Zhao et al., 2011), NaV_3_O_8_ (Zhou et al., 2013)] have been used as anode materials. Nonetheless, these ARLB anodes have problems such as low capacity, poor cycle stability, and low rate performance. In recent times, the NASCION LiTi_2_(PO_4_)_3_ anode for ARLBs has shown great potential because it has a three-dimensional channel and a stable structure (Huang et al., 2015). A NASCION-type structure is marked by an open framework, which accelerates the diffusion of Li^+^ ions in crystal. TiO_6_ octahedra and PO_4_ tetrahedra comprise the LiTi_2_(PO_4_)_3_ framework unit. The TiO_6_ octahedron is connected to the PO_4_ tetrahedron, and the structure contains a large gap channel (Vidal-Abarca et al., 2012). Wessells et al. (2011) fabricated LiTi_2_(PO_4_)_3_ and demonstrated its potential as an ARLB anode. Luo and Xia (2009) reported a supercapacitor with LiTi_2_(PO_4_)_3_ coated with carbon as the negative electrode, delivering a discharge capacity of 30 mAh g^−1^.

However, the electrochemical performance of a LiTi_2_(PO_4_)_3_-based electrode must be increased to meet the requirements of practical applications. Carbon coating, size reduction, and the introduction of a conductive agent are efficient strategies for boosting performance (Sun et al., 2015). Moreover, lattice doping is available as a means of raising anode performance (Mao et al., 2019). Wang et al. (2017) partly replaced F^−^ on a ${\text{PO}}_{4}^{3-}$ site for LiTi_2_(PO_4_)_3_ as an ARLB anode, with an energy density of 43.7 Wh Kg^1^. Liang et al. (2019) prepared Ga-doped LiTi_2_(PO_4_)_3_ film via a hydrothermal method and found favorable capacity and cycling performance. Doping with anions such as F^−^ and Cl^−^ can promote the electrokinetics of electrodes by influencing electron configuration, further increasing the electrical and ionic conductivity (Yue et al., 2013; Qi et al., 2015). In this paper, a Cl^−1^ doping strategy was employed to boost the lithium storage performance of LiTi_2_(PO_4_)_3_/C. LiTi_2_(PO_4_)_3−x_Cl_3x_/C composites were successfully prepared with a facile sol-gel method. The lithium ion storage performance of anion-doped composites was systematically studied.

## Experimental

### Synthesis

**Figure 1:**
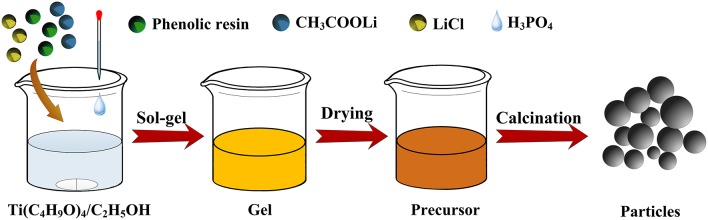
Schematic illustration of the preparation process of the composites.

All analytically pure chemical reagents were directly used without further treatment. The synthesis process of the materials is illustrated in Figure 1. Pristine LiTi_2_(PO_4_)_3_/C was synthesized via a sol-gel technique as follows. Briefly, 1.7921 g of Ti(OC_4_H_9_)_4_ was added to 20 mL of C_2_H_5_OH, and the Ti(OC_4_H_9_)_4_/C_2_H_5_OH solution was kept at room temperature with magnetic stirring. Then, 0.2659 g of CH_3_COOLi·2H_2_O and 0.1700 g of phenolic resin were dissolved in the mixture, sequentially. Following this, 0.8924 g of concentrated H_3_PO_4_ was added into 15 mL of C_2_H_5_OH and added dropwise to the above mixed solution. The mixed solution was reacted in a sealed beaker at 60°C for 2 h with magnetic stirring. When the reaction was complete, the solution was evaporated at 80°C to obtain a precursor. Finally, the precursor was calcined at 750°C for 5 h in an Ar atmosphere; the prepared LiTi_2_(PO_4_)_3_/C was named LCl. Moreover, LiTi_2_(PO_4_)_3−*x*_Cl_3*x*_/C (*x* = 0.05, 0.10, and 0.15) composites were synthesized by partially replacing ${\text{PO}}_{4}^{3-}$ with Cl^−^ in stoichiometry with LiCl. The corresponding LiTi_2_(PO_4_)_3−x_Cl_x_/C composites were denoted as LCl-05, LCl-10, and LCl-15.

### Characterizations

X-ray powder diffraction (XRD, D/MAX2500PC, Rigaku) was carried out to analyze the crystalline phase of the composites. A scanning electron microscope (S-4800, Japan) was used to investigate the morphology of the samples and cycled electrodes. Cycled electrodes were obtained after disassembling the cells, washing them with deionized water, and drying them in an oven. A thermal analyzer (TG50, Shimadzu) was used for thermogravimetric analysis (TGA) of the samples.

### Electrochemical Measurements

The electrodes were prepared as follows. The electrodes consisted of an intermixture of acetylene black, active substance, and PTFE (mass ratio: 1:1:8). The intermixture was pressed on a steel mesh with a radius of 7 mm. In cells, LiMn_2_O_4_, the synthesized samples, a saturated Li_2_SO_4_ solution, and glass fiber were used as the cathode, anode, electrolyte, and separator, respectively. A galvanostatic charge-discharge test for the cells was executed on a CT2001A testing system. Rate performance was evaluated at 0.2, 0.5, 1, 2, 5, 8, 10, 15, and 1°C, respectively. The cycling performance was recorded under 5°C for 1,000 cycles. Cyclic voltammetry and electrochemical impedance spectroscopy were performed to explore the lithium ion storage performance of Li^+^ ions using a CHI660E electrochemical workstation (Chenhua, Shanghai). Cyclic voltammetry tests were executed with a voltage range of 0–1.85 V. Electrochemical impedance spectroscopy tests for electrochemically activated cells were conducted with a frequency of 10^5^-10^−2^ Hz and an amplitude of 5 mV.

## Results and Discussion

**Figure 2:**
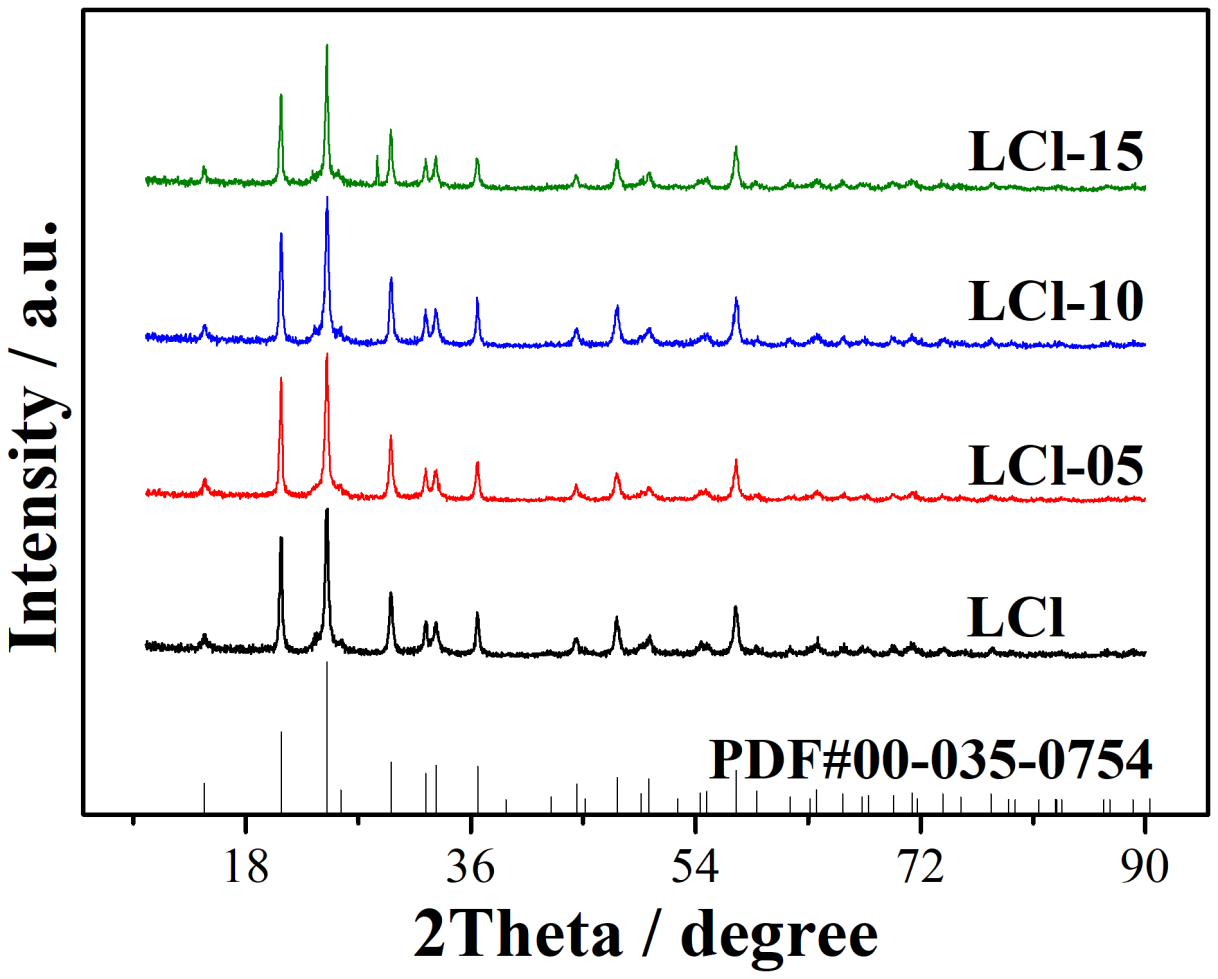
XRD patterns of NaTi_2_(PO_4_)_3−x_Cl_3x_/C (x = 0.0, 0.05, 0.10, and 0.15) composites.

The XRD patterns of the composites are presented in Figure 2. As shown, the main characteristic peaks of all composites were similar and exactly matched those of the NASICON LiTi_2_(PO_4_)_3_ with an R-3c space group (JCPDS#35-0754). This demonstrated that doping with small amount of Cl had no effect on the main structure of LiTi_2_(PO_4_)_3_. In addition, LCl-15 showed an impurity peak at 28.4 degrees, which demonstrated that a high Cl content slightly influenced the crystal structure of Cl-doped composites.

**Figure 3:**
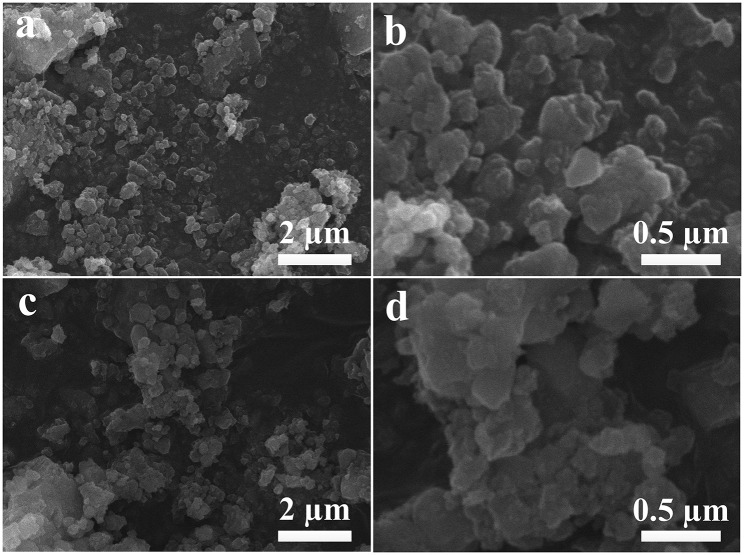
SEM images of LCl **(a,b)** and LCl-10 **(c,d)** at different magnifications.

Representative SEM images of LCl and LCl-10 at various magnifications are displayed in Figure 3. As displayed, both materials were constituted by tiny primary particles at a nanometer scale. In addition, there was some agglomeration resulting from the high temperatures reached during the synthesis process. As for the morphology of the LCl-10 composite, it exhibited no obvious differences compared to pristine LCl, further implying that Cl doping had no obvious impact on the composite morphology.

**Figure 4:**
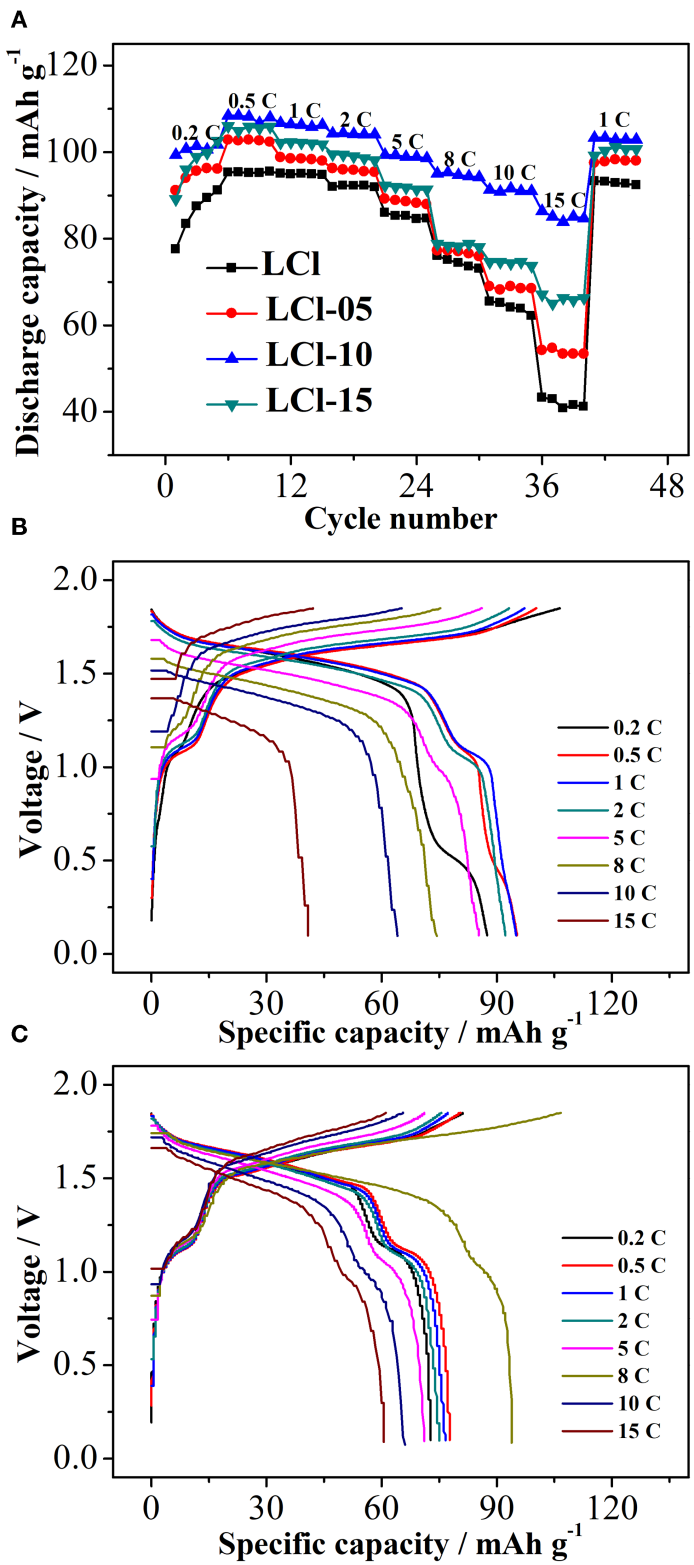
Rate performance of all composites **(A)**, charge-discharge curves for LCl **(B)** and LCl-10 **(C)** at different rates.

The rate performance of the composites is shown in Figure 4A. Serious electrochemical polarization in a large current resulted in a decrease in the discharge capacity of all composites as the rate increased. The discharge capacity of Cl-doped LiTi_2_(PO_4_)_3_ composites, particularly LCl-10, was increased compared to the original LiTi_2_(PO_3_)_4_/C, indicating an improvement with Cl-doped anode materials. The difference in discharge capacity between Cl-doped LiTi_2_(PO_4_)_3_ composites and the original LiTi_2_(PO_3_)_4_/C became more obvious as the rate was increased. LCl-10 delivered a discharge capacity of 108.5 and 85.5 mAh g^−1^ at 0.5 and 15°C, respectively, an increase of 13.1 and 44.3 mAh g^−1^ compared to pristine LCl. Charge-discharge curves for the LCl and LCl-10 composites at disparate rates are displayed in Figures 4B,C, respectively. There were two voltage plateaus for LCl and LCl-10, at about 1.0 and 1.5 V. The voltage plateaus of LCl-10 were wider and more stable compared to those of LCl, revealing the outstanding electrochemical performance of LCl-10.

**Figure 5:**
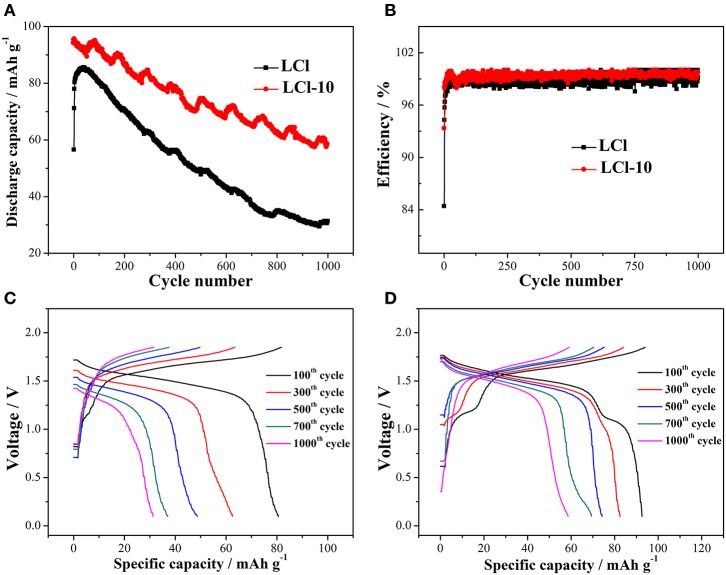
Cycling performances **(A)** and coulombic efficiency **(B)** for LCl and LCl-10 at 5°C for 1,000 cycles, charge-discharge curves for LCl **(C)** and LCl-10 **(D)** after different cycles.

The cycling properties of LCl and LCl-10 composites were studied at 5°C for 1,000 cycles, and the results are shown in Figure 5A. The discharge capacity of both composites gradually increased during the first several cycles due to electrode activation. The maximal discharge capacity of LCl-10 (95.27 mAh g^−1^) was higher than that of LCl (84.89 mAh g^−1^). The discharge capacity of LCl-10 was maintained at 61.3% after 1,000 cycles, which was 25.1% higher than that of LCl (36.2%). Figure 5B shows that the coulombic efficiencies of LCl and LCl-10 were in the vicinity of 100%, revealing that the cells had almost no self-discharge. The charge-discharge curves for LCl and LCl-10 after various cycles are shown in Figures 5C,D, respectively. The relatively wider and more stable voltage plateau for LCl-10 demonstrated good electrochemical performance and stability, unveiling the remarkable cycling properties of LCl-10.

**Figure 6:**
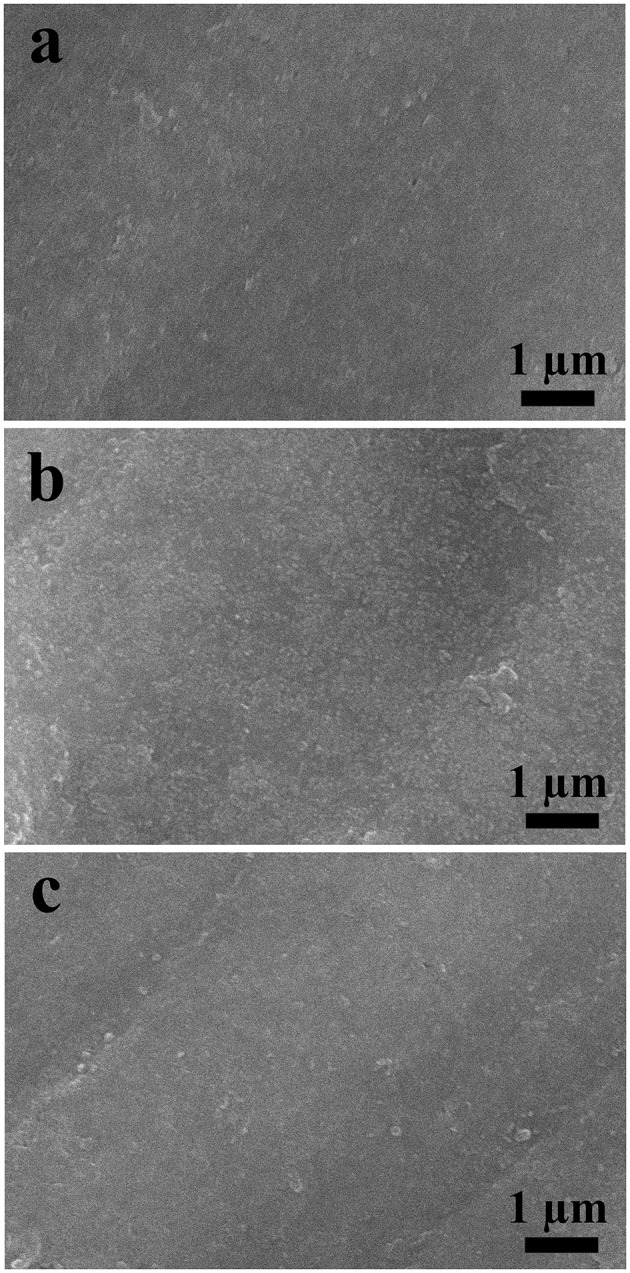
SEM images of LCl-10 anodes after 100 **(a)**, 500 **(b)**, and 1,000 **(c)** cycles at a rate of 5°C.

In order to study the structural stability of the electrode after multiple cycles, the electrode morphology was analyzed by SEM. The results for LCl-10 electrodes after 100, 500, and 1,000 cycles are shown in Figure 6. As can be seen, the LCl-10 electrode surface was smooth after 100 cycles, and the morphology remained stable even after 1,000 cycles, without apparent dissolution. This demonstrated that LCl-10 had excellent structural stability after a long period of charging and discharging.

**Figure 7:**
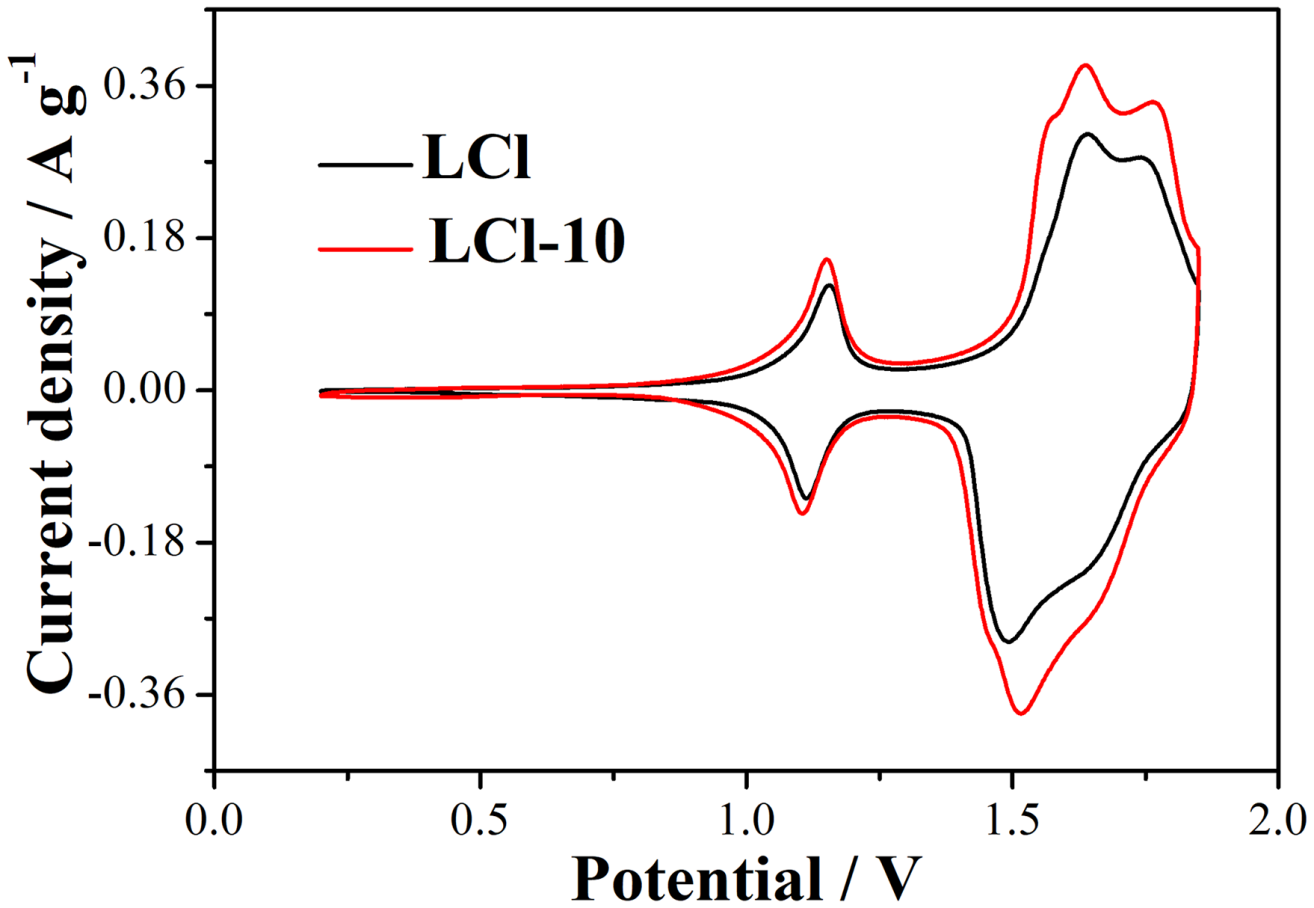
Cyclic voltammetry curves of the cells using LCl and LCl-10 at a scan rate of 0.5 mV s^−1^.

Cyclic voltammetry tests of the cells for LCl and LCl-10 were carried out to study the electrochemical kinetics, and the results are shown in Figure 7. As can be observed, both LCl and LCl-10 had two obvious current peaks. Further, the current density of LCl-10 was higher than the corresponding current density of LCl. The higher current density and narrower peak potential difference of LCl-10 illustrated the excellent electrochemical property of LCl-10. The anodic and cathodic peak current density of LCl-10 at 1.6 V reached 0.38 and 0.38 A g^−1^, respectively, with an obvious improvement compared to LCl (0.30 and 0.30 A g^−1^). The peak potential difference of LCl-10 with a value of 0.12 V was smaller than that of LCl (0.15 V), indicating better electrochemical reversibility of the modified electrode.

**Figure 8:**
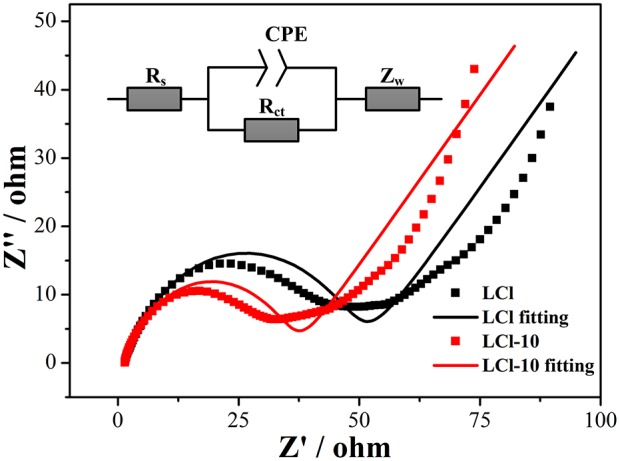
Electrochemical impedance spectra and fitting results of the assembled cell for LCl and LCl-10.

An electrochemical impedance spectroscopy test was implemented to investigate the effect of Cl doping on the electrochemical kinetics. Nyquist plots and fitting results for LCl and LCl-10 are shown in Figure 8. The Nyquist plots were characterized by an intercept, a semicircle, and an inclined line at high, middle, and low frequencies, parallel to ohmic resistance (R_s_) including the electrolyte and electrode, charge transfer resistance (R_ct_) linked with the electrochemical reaction, and Warburg impedance (Z_w_) related to the migration of Li ions, respectively. As can be seen, the electronic conductivity of LCl-10 did not change significantly compared to that of LCl. This indicated that Cl doping caused no obvious change in the electronic conductivity of the material. This was due to the improvement in electronic conductivity from the introduction of carbon. LCl-10 delivered an R_ct_ value of 30.0 Ω, which was less than that of LCl (43.9 Ω), illustrating that the Cl doping had a positive effect on the electrochemical kinetics.

## Conclusions

LiTi_2_(PO_4_)_3_/C composites doped with chlorine on phosphate spots were successfully synthesized with a sol-gel technique. Chlorine doping did not significantly impact the main structure and morphology of LiTi_2_(PO_4_)_3_/C. Of all the samples, LCL-10 composites showed the optimum rate performance, and their advantages were more obvious at a higher rate. LCl-10 had a discharge capacity of 108.5 and 85.5 mAh g^−1^ at 0.5 and 15°C, respectively, which was 13.1 and 44.3 mAh g^−1^ higher than those of LCl. Moreover, the discharge capacity of LCl-10 was maintained at 61.3% after 1,000 cycles at 5°C, which was 25.1% higher than that of LCl (36.2%). In a word, LiTi_2_(PO_4_)_3_/C doped with an appropriate amount of chlorine is a potential anode for high-performance ARLBs.

## Data Availability Statement

All datasets generated for this study are included in the article/supplementary material.

## Author Contributions

HL: carried out experiments and wrote the manuscript. YT: carried out experiments. ZX: performed analyzed experimental results. PW: designed experiments. ZL: revised the manuscript.

## Conflict of Interest

The authors declare that the research was conducted in the absence of any commercial or financial relationships that could be construed as a potential conflict of interest.
